# The Transmembrane Adaptor Protein SIT Inhibits TCR-Mediated Signaling

**DOI:** 10.1371/journal.pone.0023761

**Published:** 2011-09-21

**Authors:** Börge Arndt, Tina Krieger, Thomas Kalinski, Anja Thielitz, Dirk Reinhold, Albert Roessner, Burkhart Schraven, Luca Simeoni

**Affiliations:** 1 Institute of Molecular and Clinical Immunology, Otto-von-Guericke University Magdeburg, Magdeburg, Germany; 2 Institute of Pathology, Otto-von-Guericke University Magdeburg, Magdeburg, Germany; 3 Clinic of Dermatology and Venerology, Otto-von-Guericke University Magdeburg, Magdeburg, Germany; Oklahoma Medical Research Foundation, United States of America

## Abstract

Transmembrane adaptor proteins (TRAPs) organize signaling complexes at the plasma membrane, and thus function as critical linkers and integrators of signaling cascades downstream of antigen receptors. We have previously shown that the transmembrane adaptor protein SIT regulates the threshold for thymocyte selection. Moreover, T cells from SIT-deficient mice are hyperresponsive to CD3 stimulation and undergo enhanced lymphopenia-induced homeostatic proliferation, thus indicating that SIT inhibits TCR-mediated signaling. Here, we have further addressed how SIT regulates signaling cascades in T cells. We demonstrate that the loss of SIT enhances TCR-mediated Akt activation and increased phosphorylation/inactivation of Foxo1, a transcription factor of the Forkhead family that inhibits cell cycle progression and regulates T-cell homeostasis. We have also shown that CD4^+^ T cells from SIT-deficient mice display increased CD69 and CD40L expression indicating an altered activation status. Additional biochemical analyses further revealed that suppression of SIT expression by RNAi in human T cells resulted in an enhanced proximal TCR signaling. In summary, the data identify SIT as an important modulator of TCR-mediated signaling that regulates T-cell activation, homeostasis and tolerance.

## Introduction

Transmembrane adaptor proteins (TRAPs) are a group of molecules involved in immune cell activation [1]. Among these, LAT, PAG/Cbp, NTAL/LAB and LIME are mainly localized in the lipid rafts; while TRIM, SIT and LAX are excluded from the raft fraction of the plasma membrane (reviewed in 1).

The common characteristic of TRAPs is the presence of several tyrosine-based signaling motifs (TBSMs) within their cytoplasmic tails. These are phosphorylated by protein tyrosine kinases of the Src and Syk families, thereby, enabling the TRAPs to recruit SH2-containing cytoplasmic signaling and/or effector proteins to the plasma membrane. Thus, TRAPs function as important regulators of signal transduction, connecting signal transducing cell-surface receptors with downstream cellular signaling pathways [1].

During the last years, several groups have generated knockout mice to investigate the *in vivo* function of these molecules. The data demonstrate that TRAPs play an important role in the regulation of immune-cell homeostasis. For example, LAT-deficient mice display a severe alteration of the immune system and T-cell development is completely blocked at the DN3 stage [2]. NTAL-deficient mice develop a spontaneous autoimmune disease characterized by splenomegaly, elevated levels of anti-double-stranded DNA autoantibodies, and deposition of immune complexes within the glomeruli [3]. Moreover, LAX-deficient mice display hyperactive T, B and mast cells [4], [5].

We have shown that SIT-deficient mice have altered thymic selection and decreased numbers of peripheral naïve T cells [6]–[8]. Moreover, T cells from SIT^−/−^ mice also display hyperresponsiveness to CD3 stimulation and undergo enhanced lymphopenia-induced expansion [6], [7]. Finally, we showed that the clinical course of experimental autoimmune encephalomyelitis (EAE) is more severe in SIT^−/−^ mice [6]. Thus, these data suggest that SIT plays an important role in T-cell homeostasis and in the establishment of tolerance.

In the present study, we extended our previous investigations on the function of SIT. We focused particularly on how SIT regulates signaling in T cells. We provide further evidence that SIT negatively regulates proximal TCR signaling and TCR-mediated phosphorylation of Akt *in vitro*. We additionally show that the phosphorylation/inactivation of Foxo1, a transcription factor downstream of Akt that inhibits cell cycle progression and regulates T-cell homeostasis [9], [10], is significantly enhanced in SIT^−/−^ T cells. Moreover, CD4^+^ T cells within the secondary lymphoid organs of SIT^−/−^ mice show signs of mild hyperactivation. In summary, our studies demonstrate that SIT is an important inhibitory molecule of T-cell activation.

## Methods

### Human Ethics

Approval for these studies involving the analysis of TCR-mediated signaling in human T cells was obtained from the Ethics Committee of the Medical Faculty at the Otto-von-Guericke University, Magdeburg, Germany with the permission number [107/09]. Informed consent was obtained in writing in accordance with the Declaration of Helsinki.

### Animal Ethics

All experiments were performed with samples taken from euthanized animals in accordance with the German National Guidelines for the Use of Experimental Animals (Animal Protection Act, Tierschutzgesetz, TierSchG). Animals were handled in accordance with the European Communities Council Directive 86/609/EEC. All possible efforts were made to minimize animal suffering and the number of animals used.

### Mice

SIT^−/−^ mice were previously described [6]. The mice were maintained in a pathogen free condition.

### Flow cytometry

Single cell suspensions were prepared from spleen. Subsequently, 1×10^6^ cells were stained with different monoclonal antibodies for 15 min at 4°C, washed and then analyzed on a FACSCalibur using the CellQuest software (Becton Dickinson). Antibodies were purchased from BD Pharmingen. Anti-CD40L (MR1) Abs was from eBioscience.

The analysis of Annexin V, Bcl-2, and BrdU was performed as previously described [7].

### Cell culture

Peripheral blood mononuclear cells were isolated by Ficoll gradient (Biochrom) centrifugation of heparinized blood collected from healthy volunteers. Human T cells were further purified by non-T cell depletion using the Pan T cell isolation kit II (Miltenyi Biotec). The purity of T cells, determined by flow cytometry, was usually more then 96%. Cells were maintained in RPMI 1640 medium containing 10% FBS, stable L-glutamine, and 1000 U/ml penicillin/streptomycin at 37°C with 5% CO_2_ over night before transfection. Jurkat T cells (ATCC) were maintained in RPMI 1640 medium supplemented with 10% FBS (PAN) and stable L-glutamine at 37°C with 5% CO_2_.

### RNA interference, plasmids and cell transfection

The following SIT siRNA duplex containing 25 nucleotides were purchased from Invitrogen: 5′-GGC UGC AGA GGA GGU GAU GUG CUA U-3′, 5′-GGG CUG UGA CGC UGC UAU UUC UCA U-3′ and 5′-GCC AGA CCA GCA GGA UCC AAC UCU U-3′. As negative control we used a Renilla Luciferase siRNA duplex from Invitrogen. To achieve efficient SIT downregulation, primary human T cells (3×10^6^) were transfected with a mix of siRNA (120 nM for each duplex) using the Nucleofection Kit (Amaxa) according to the manufacturer's instruction or the Gene Pulser Xcell (Bio-Rad) as previously described [11]. Cells were harvested 72 h after electroporation. 20×10^6^ Jurkat T cells were resuspended in 0.4 ml of PBS containing Ca^2+^ and Mg^2+^ and incubated with a mix of siRNA (200 nM for each duplex). Cells were transfected using Gene Pulser II (Bio-Rad) set at 230 mV, 950 µF. Cells were cultured for 48 h after electroporation.

### Cell stimulation, Immunoblotting, and immunoprecipitation

Purified mouse CD4^+^ T cells were stimulated at 37°C with 5 µg/ml soluble anti-mouse CD3ε (145-2C11; BD Biosciences) without cross-linking for the indicated time points. Human T cells were stimulated at 37°C by using OKT3 mAb without cross-linking for the indicated time points. Cell lysates were prepared and analyzed as previously described [12]. Anti-pS^473^Akt (PKB), anti-pY^319^ZAP70, anti-pY^191^LAT, anti-pS^256^Foxo1, (all from Cell Signaling Technology), anti-pY^783^PLCγ-1, CD3ε (Santa Cruz Biotechnology), anti-pY^145^SLP-76 (BD Biosciences), anti-β-actin (clone AC-15) (Sigma-Aldrich), anti-p-Tyr (4G10) (Upstate Biotechnology), anti-ZAP-70 (Transduction Laboratories), and anti-SIT [13] antibodies were used for Western blotting. The intensity of the detected bands was acquired using the Kodak Image station 2000R and analysis was performed using ID Image software (Kodak).

In immunoprecipitation experiments, 10×10^6^ Jurkat T cells were either left unstimulated or stimulated with 5 µg/ml purified OKT3 mAb without cross-linking for 5 min at 37°C. Cell lysates were immunoprecipitated with agarose-conjugated CD3ζ (Santa Cruz Biotechnology) (Santa Cruz Biotechnology) antibody followed by recombinant protein G-Sepharose 4B (Zymed Laboratories Inc.) at 4°C overnight. After washing, CD3ζ immunoprecipitates were resolved by SDS-PAGE, transferred to a membrane, and analyzed by immunoblotting with the indicated antibodies.

### Statistics

Statistical analyses were performed using GraphPad Prism (GraphPad Software Inc., San Diego, CA). *p* values were determined by an unpaired two-tailed Student's *t* test.

## Results

### Loss of SIT results in an enhanced activation of the TCR-mediated Akt-Foxo pathway

**Figure 1:**
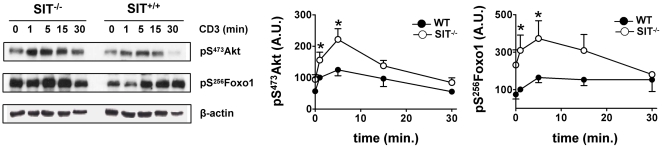
Enhanced phosphorylation of Akt and Foxo1 in SIT^−/−^ CD4^+^ T cells. Purified splenic CD4^+^ T cells from 3–4 month-old SIT^−/−^ or WT mice were stimulated with non-crosslinked CD3 (clone 145-2C11) mAb for the indicated times. Samples were analyzed by Western blotting using the indicated Abs. The phosphorylated Akt and Foxo1 bands were quantified using the ImageQuant software and values were normalized to the corresponding β-actin signal. Graphs show the phosphorylation levels of Akt and Foxo1 shown as of arbitrary units ± SEM of at least 3 independent experiments. Statistical significance * p<0.05.

Recently, we have shown that SIT is an important modulator of T-lymphocyte functions. SIT negatively regulates selection processes within the thymus, peripheral T-cell activation and T-cell homeostasis [6]–[8]. Moreover, SIT-deficient mice are more susceptible to the development of EAE, a mouse model of Multiple Sclerosis [6]. As the data suggest that SIT plays an inhibitory role in T cells, we have next attempted to investigate whether SIT influences TCR-mediated signaling. However, we have found that SIT-deficient T cells develop sensory adaptation and are refractory to stimulation upon extensive crosslinking of the TCR/CD3 complex [7], Therefore, we used an alternative stimulation condition that induces only a minimal crosslinking of the antigen receptor on T cells, thus better mimicking the physiological situation. Under these conditions of stimulation, we found that SIT-deficient T cells show enhanced Akt phosphorylation (figure 1). We next investigated which intracellular signaling pathways might be affected by the augmented Akt activation in SIT-deficient T cells. We have shown that the loss of SIT results in an enhanced T-cell proliferation [6], [7]. Therefore, as a putative target of Akt, we chose Foxo1, a member of the Forkhead family of transcription factors, which is implicated in cell cycle regulation [9]. One of the known functions of Foxo is to activate the transcription of the cell cycle inhibitor p27^kip1^. Members of the Foxo family are phosphorylated by Akt. Phosphorylation by Akt results in the sequestration of Foxo1 into the cytosol, thus preventing p27^kip1^ transcription and cell cycle inhibition. Although well characterized in other cellular systems, it is poorly understood how the Akt-Foxo pathway is activated in T cells. Recently, it has been shown in T cells that Vav1 activates the PI3K-Akt-Foxo1 pathway, thus regulating cell cycle progression [14]. Loss of Vav1 resulted in reduced activation of Akt, blunted Foxo1 phosphorylation, and cell cycle arrest. Conversely to Vav1^−/−^ T cells, SIT-deficient T cells are hyperproliferative and display enhanced Akt activation. Thus, we expected to observe an enhanced phosphorylation of Foxo1 in SIT^−/−^ T cells. As shown in figure 1, we found that the level of phospho-Foxo1 is indeed higher in lysates from SIT-deficient CD4^+^ T cells than in wild type cells. These results suggest that SIT inhibits TCR-mediated Akt activation and Foxo1 phosphorylation, thus likely negatively regulating T-cell proliferation.

### Suppression of SIT expression enhanced proximal TCR signaling

**Figure 2:**
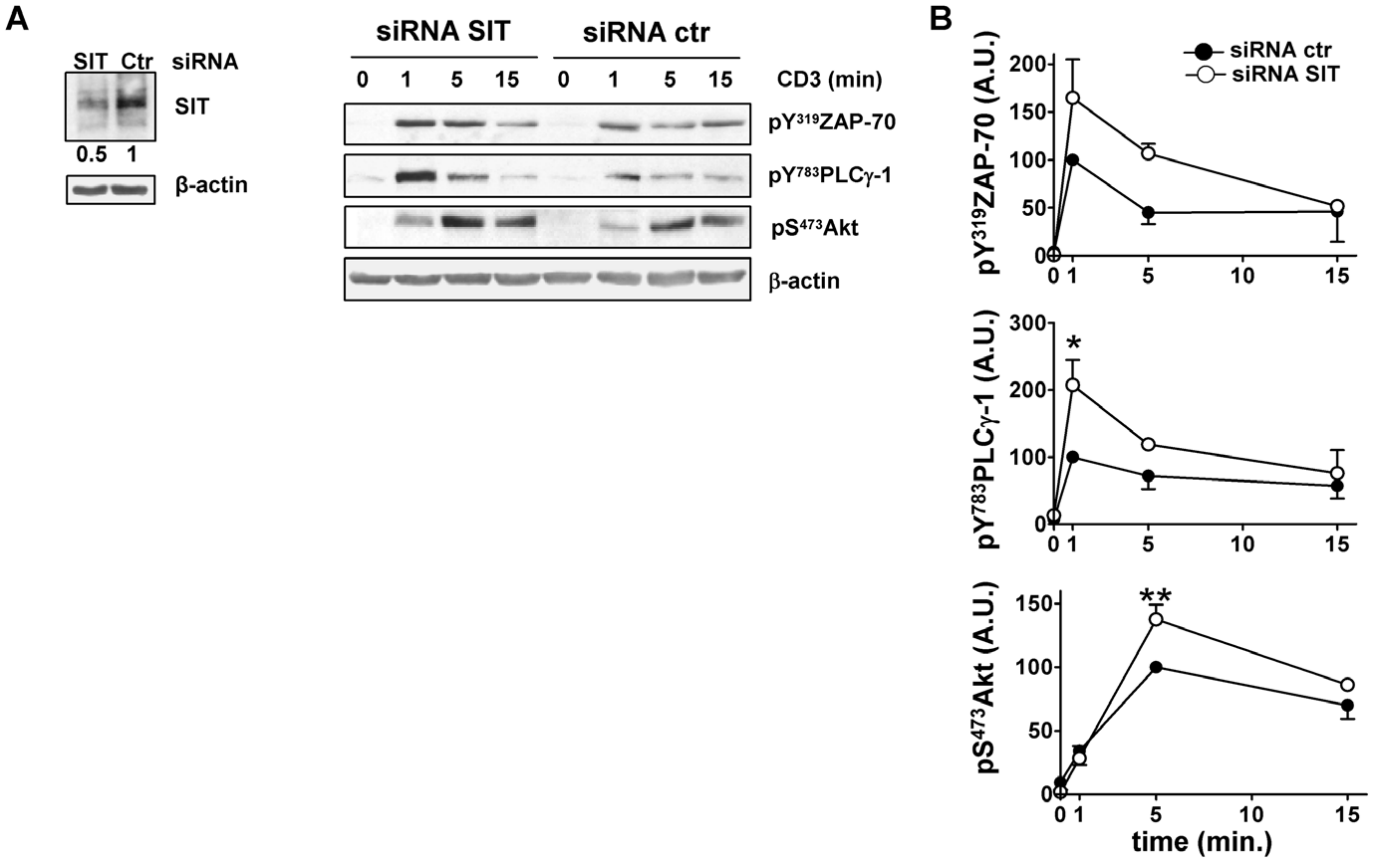
Downregulation of SIT results in an enhanced TCR-mediated signaling in primary human T cells. A) Primary human T cells were transfected with control or SIT-specific siRNA and 72 h later were stimulated with non-crosslinked CD3 (clone OKT3) mAb for the indicated times. Cell lysates were analyzed by immunoblotting using phosphospecific antibodies for ZAP-70, PLCγ-1, and Akt. Immunoblots verifying SIT downregulation are shown. B) Phosphorylated bands were quantified using the ImageQuant software and values were normalized to the corresponding β-actin signal. Graphs show the phosphorylation levels of ZAP-70, PLCγ-1, and Akt shown as of arbitrary units ± SEM of 2, 4, and 6 independent experiments, respectively. Statistical significance in B) * p<0.05 and **p<0.01.

As mentioned above, SIT-deficient mouse T cells develop compensatory mechanisms that alter proximal TCR signaling [7]. Therefore, the enhanced phosphorylation of Akt observed in SIT-deficient T cells could be due to the altered status of SIT-deficient mouse T cells. To confirm that SIT regulates Akt activation, we performed RNA interference in primary human T cells. The results shown in figure 2 demonstrate that, similar to mouse T cells, downregulation of SIT in human primary T cells results in an enhanced TCR-mediated Akt phosphorylation.

Collectively, these data suggest that SIT regulates T-cell activation likely by inhibiting proximal TCR signaling events leading to Akt activation. Therefore, we next analyzed proximal TCR signaling in primary human T cells upon downregulation of SIT. As shown in figure 2, we found that the phosphorylation of both ZAP-70 and PLCγ-1 was enhanced. Thus, it appears that SIT suppresses proximal TCR signaling.

**Figure 3:**
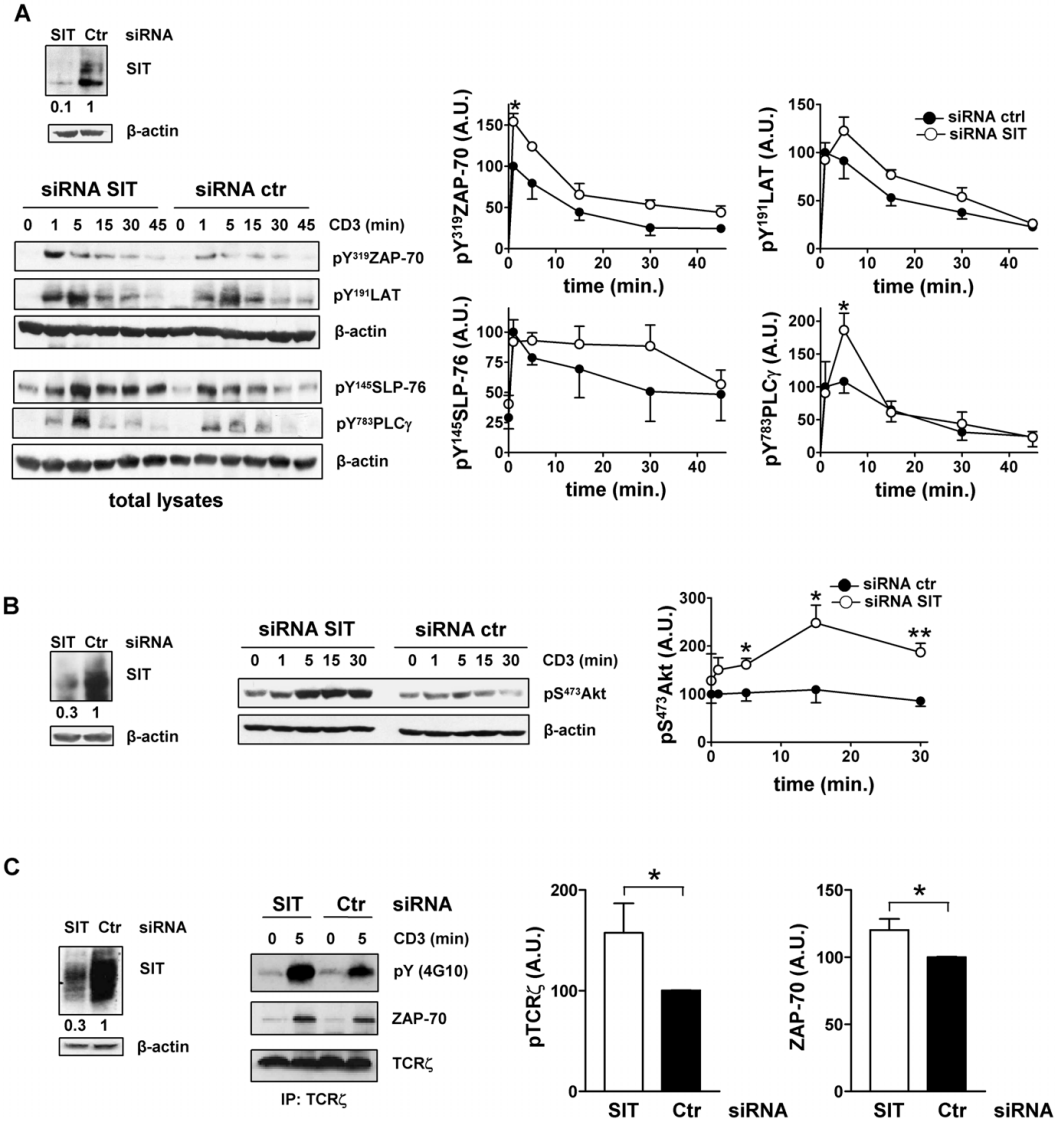
Suppression of SIT expression results in an enhanced proximal TCR signaling. siRNA duplex for SIT or control were introduced in Jurkat T cells by electroporation. Cells were stimulated 48 h after transfection with non-cross-linked CD3 Ab for the indicated times. Total cell lysates (A–B) or TCRζ immunoprecipitates (C) were prepared and analyzed by Western blotting using the indicated Abs. The data are representative of at least 3 independent experiments. Immunoblots verifying SIT downregulation are shown. Equal loading is shown by reprobing immunoblots with antibodies specific for β-actin (A–B) or TCRζ (C). The phosphorylated bands were quantified using ImageQuant software and values were normalized to the corresponding β-actin signal. Graphs show the phosphorylation levels shown as of arbitrary units ± SEM of at least 3 independent experiments. Statistical significance * p<0.05 and **p<0.01.

In primary human T cells, the downregulation efficiency of SIT is approximately 50% (figure 2A). In order to obtain a more efficient suppression of SIT, we used the Jurkat T-cell line. As shown in figure 3, we were able to downregulate up to 70–90% of SIT in Jurkat T cells. Therefore, we used this well-characterized T-cell line to further analyze the role of SIT on proximal TCR signaling. The data presented in figure 3A show that, similar to the downregulation of SIT in primary human T cells, also suppression of SIT in Jurkat T cells results in an enhanced proximal TCR signaling. SIT-depleted Jurkat T cells showed an augmented TCR-mediated phosphorylation of ZAP-70, LAT, SLP-76 and PLCγ-1 (figure 3A). We additionally found that suppression of SIT strongly enhanced Akt phosphorylation upon CD3 stimulation even in a system where Akt is already constitutively activated (figure 3B) [15], [16].

To further elucidate the mechanism responsible for the enhanced proximal TCR signaling, we investigated TCRζ phosphorylation and TCRζ/ZAP-70 association. In agreement with the enhanced phosphorylation of ZAP-70, LAT, SLP-76, and PLCγ-1, the phosphorylation of TCRζ was also enhanced upon suppression of SIT expression (figure 3C). Additionally, analysis of immunoprecipitated TCRζ showed that the association with ZAP-70 was also increased in SIT dim T cells (figure 3C). Thus, these data suggest that suppression of SIT expression enhanced proximal TCR signaling at the level of TCRζ phosphorylation.

### Increased proportion of activated CD4^+^ T cells in aged SIT-deficient mice

**Figure 4:**
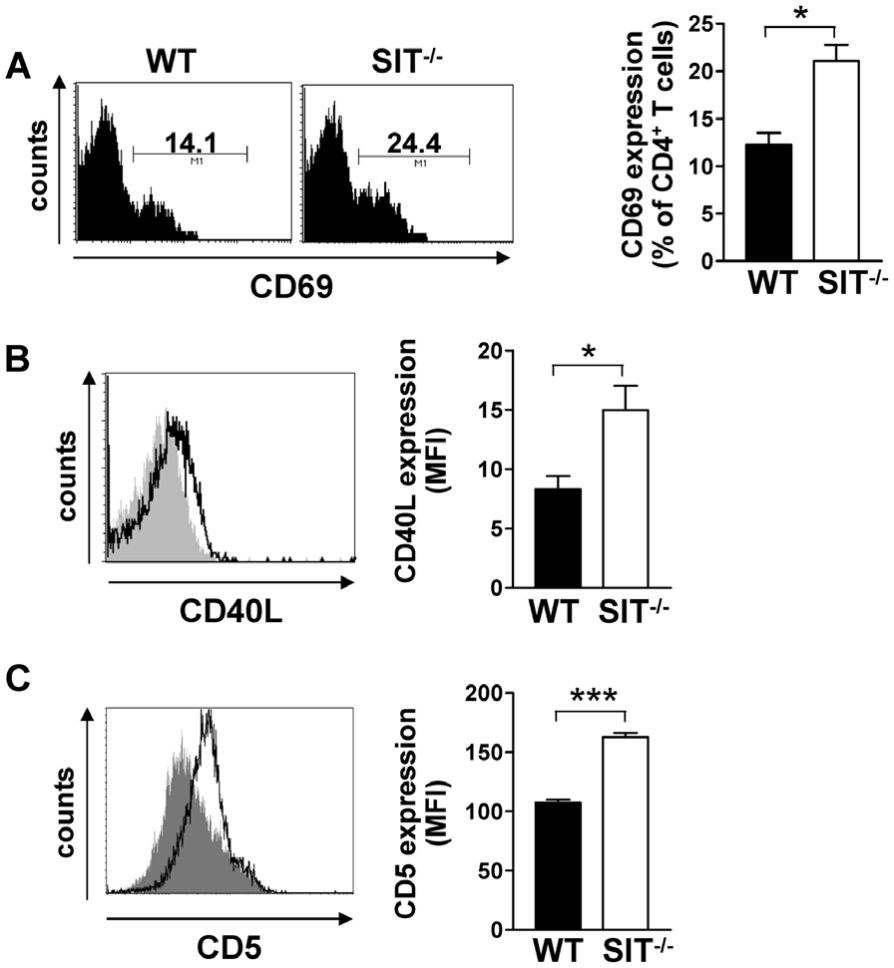
Analysis of activation markers in SIT^−/−^ mice. A) Splenic CD4^+^ T cells from 1 year-old SIT-deficient and wild-type mice were analyzed for CD69 expression. Numbers within histograms indicate the percentage of cells. One representative profile per group is shown. B) Comparison of CD40L expression on CD4^+^ T cells isolated from SIT-deficient (empty histogram) and wild-type (filled histogram) mice. Mean fluorescent intensity (MFI) from one representative mouse per group is indicated. C) Comparison of CD5 expression on CD4^+^ T cells isolated from SIT-deficient (empty histogram) and wild-type (filled histogram) mice. Bar graphs in A), B), and C) represent statistical analyses of CD69, CD40L, and CD5 expression, respectively. Data derive from at least 5 mice per group. Statistical significance * p<0.05 and ***p<0.0001.

**Figure 5:**
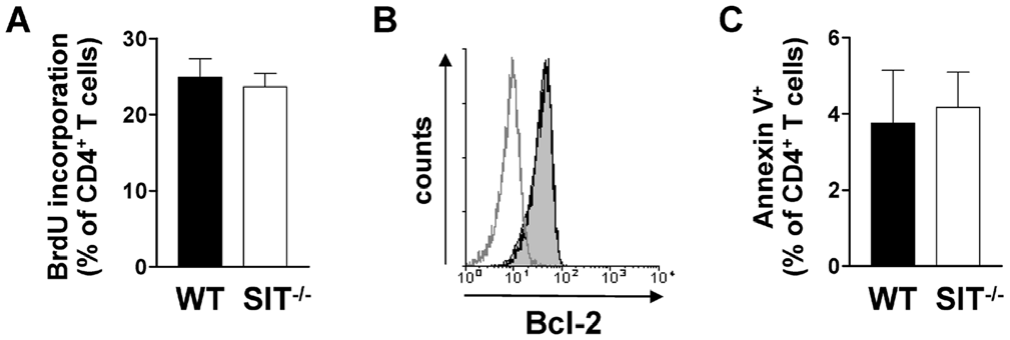
Normal survival and steady-state proliferation of SIT-deficient CD4^+^ T cells. Lymph node cells were isolated and stained with CD4 and CD8 mAbs. A) BrdU incorporation was measured for CD4^+^ T cells. Bar graphs indicate mean values ± SEM from 3 SIT^−^/^−^ and 5 SIT^+^/^+^ mice. B) Intracellular Bcl-2 expression in CD4^+^ T cells from SIT^−^/^−^ (empty histogram) and SIT^+^/^+^ (filled histogram) mice. Isotype control is shown as a gray line. C) Annexin V expression on CD4^+^ T cells. Bar graphs indicate mean values ± SEM from 3 SIT^−^/^−^ and 3 SIT^+^/^+^ mice.

The PI3K-Akt pathway regulates a variety of cellular processes including proliferation and apoptosis. Therefore, we next investigated whether SIT^−/−^ mice developed any dysfunction within the lymphocyte compartment. We found that CD4^+^ T cells from aged SIT^−/−^ mice showed a modest but consistent increase in the expression of CD69 and CD40L (figure 4A, 4B). Moreover, these cells also express increased levels of CD5 (figure 4C), which we have previously shown to be altered also in thymocytes and CD8^+^ T cells [6], [7]. We additionally analyzed the expression of a variety of other surface markers, including CD3, CD4, CD25, CD44, CD45RB, CD62L, and CD103, but we did not observe significant differences between WT and SIT-deficient mice ([6], [7], table 1, and table 2). We also did not observe significant differences in the size of secondary lymphoid organs. In fact, the numbers of total splenocytes, total splenic CD4^+^ T cells, and effector/memory CD4^+^ T cells in the spleen were not affected (table 2). We found only a modest decrease in the number of CD4^+^ T cells in lymph nodes [6]. Finally, we also did not observe significant differences in BrdU incorporation (figure 5A), in the intracellular expression of the antiapoptotic molecules Bcl-2 (figure 5B), and in Annexin V expression (figure 5C) on *ex vivo* isolated CD4^+^ T cells from SIT-deficient mice. Thus, these data indicate that loss of SIT results in a partial alteration of the activation status of CD4^+^ T cells without affecting their steady-state proliferation or apoptosis. We hypothesize that the compensatory mechanism displayed by SIT-deficient mice may prevent the development of a more severe hyperactivation of T cells and lymphoproliferative diseases.

**Table 1:** T-cell subsets in lymph nodes.

Genotype	βTCR^+^CD45RB^+^	βTCR^+^CD62L^+^	CD4^+^CD25^+^	CD4^+^CD103^+^
SIT^+/+^	32.8±3.5 (n = 6)	66.3±7.0 (n = 4)	7.5±0.2 (n = 3)	2.8±0.5 (n = 10)
SIT^−/−^	27.2±1.8 (n = 6)	60.3±7.5 (n = 4)	7.6±0.6 (n = 3)	2.8±0.4 (n = 10)

% of lymph node cells ± SEM. n = number of examined mice.

**Table 2:** T-cell subpopulations in the spleen.

Genotype	Splenocytes	CD4^+^	CD4^+^CD44^hi^CD62^lo^
SIT^+/+^ (n = 8)	111.3±24.6	19.6±6.5	8.64±3.8
SIT^−/−^ (n = 8)	98.8±26.1	19.6±4.1	11.44±2.5

Number of cells in the spleen (×10^6^) ± SEM. n = number of examined mice.

## Discussion

In this study, we provide new insights into the function of the transmembrane adaptor protein SIT. We show that SIT inhibits TCR-mediated signaling. This is an important finding as the mechanism by which SIT inhibits T-cell activation was not clear. We have previously shown that overexpression of SIT suppressed transcription of an NFAT-driven reporter gene in the Jurkat T-cell line [13]. However, we did not find obvious alterations of more proximal TCR signaling events in this T-cell line upon strong stimulation of the antigen receptor. Here, we now show that SIT negatively regulates the phosphorylation/activation of crucial signaling molecules such as ZAP-70, LAT, PLCγ-1, and Akt upon weak T-cell stimulation. Potential clues to the mechanisms underlying the inhibitory effect of SIT on TCR-mediated signaling may be provided by recent unexpected observations suggesting that Grb2 amplifies TCR signaling by regulating Lck activation [17]. SIT possesses two Grb2 consensus binding motifs and indeed inducibly binds Grb2 upon TCR stimulation [13], [18]. It has been suggested that non-raft TRAPs inhibit antigen-receptor-mediated signaling by sequestering signaling molecules away from the lipid rafts [1]. Thus, we propose a scenario whereby SIT, by functioning as a dosage regulator of Grb2 at the plasma membrane, may in turn dampen proximal TCR signaling.

We have additionally uncovered a link between the transmembrane adaptor SIT and the Akt-Foxo1 pathway. Whether SIT inhibits the phosphorylation of Akt-Foxo1 directly or indirectly (i.e. by negatively regulating proximal signaling) requires further investigation. Nevertheless, our study adds new insights into the regulation of Foxo1 in T cells. We show that low affinity ligands induced Foxo1 phosphorylation/inactivation in mouse T cells and that the loss of SIT enhances the TCR-mediated inactivation of Foxo1. Thus, SIT could modulate cell cycle progression (e.g. triggered by low affinity ligands) via Foxo1. Intriguingly, similar to SIT^−/−^ mice [6], [7] Foxo1^−/−^ mice display reduced numbers of naïve cells and an accumulation of CD44^hi^ memory-like T cells [10]. It will be interesting to determine whether SIT is involved in the regulation of other members of the Foxo family such as Foxo4 and Foxo3a. In fact, the characterization of Foxo3a-deficient mice revealed a phenotype resembling that of SIT-deficient mice, such as T-cell hyperactivation, enhanced production of IL-2 and Th1 and Th2 cytokines [19].

We have previously shown that SIT regulates thymocyte selection [6], [8]. It is tempting to speculate that an altered Foxo-mediated pathway also accounts for the defects in thymic development in SIT-deficient mice. Indeed, it has been recently shown that the adaptor protein SLP-65 regulates B-cell development via Akt-Foxo3a [20]. How Foxo family members regulate lymphocyte development is not yet clear. They may be involved in the regulation of transcriptional activation of genes required in developmental processes.

Our data suggest that the loss of SIT also results in an altered function of CD4^+^ T cells. In fact, both TCR-mediated signaling and the expression levels of CD69 and CD40L are augmented. Moreover, we have previously shown that SIT-deficient T cells were hyperresponsive to CD3 stimulation, produced higher amount of cytokines *in vitro*, and more importantly, displayed a more severe clinical course of the experimentally induced autoimmunity EAE [6]. Thus, it appears that the loss of SIT generally enhances the activation status of T lymphocytes. Despite the fact that T cells are hyperactivated, SIT-deficient mice do not develop signs of chronic inflammation or lymphoproliferation. We propose that the development of compensatory mechanisms in SIT-deficient mice [7] could serve to prevent autoimmunity or chronic inflammatory processes, thus preserving immune functions. However, whether sensory adaptation is completely effective is currently under investigations. In fact, we have recently found that SIT-deficient mice spontaneously develop anti-nuclear antibodies and glomerulonephritis (Arndt and Simeoni, unpublished observations).

In summary, the data presented here together with previous studies demonstrate a critical function for SIT in fine-tuning signals emanating from the TCR leading to proliferation, differentiation and tolerance and further emphasize the important role that TRAPs play in regulating homeostasis within the immune system.

## References

[1] Horejsi V, Zhang W, Schraven B (2004). Transmembrane adaptor proteins: organizers of immunoreceptor signalling.. Nat Rev Immunol.

[2] Zhang W, Sommers CL, Burshtyn DN, Stebbins CC, DeJarnette JB (1999). Essential role of LAT in T cell development.. Immunity.

[3] Zhu M, Koonpaew S, Liu Y, Shen S, Denning T (2006). Negative regulation of T cell activation and autoimmunity by the transmembrane adaptor protein LAB.. Immunity.

[4] Zhu M, Granillo O, Wen R, Yang K, Dai X (2005). Negative regulation of lymphocyte activation by the adaptor protein LAX.. J Immunol.

[5] Zhu M, Rhee I, Liu Y, Zhang W (2006). Negative regulation of Fc epsilonRI-mediated signaling and mast cell function by the adaptor protein LAX.. J Biol Chem.

[6] Simeoni L, Posevitz V, Kolsch U, Meinert I, Bruyns E (2005). The transmembrane adapter protein SIT regulates thymic development and peripheral T-cell functions.. Mol Cell Biol.

[7] Posevitz V, Arndt B, Krieger T, Warnecke N, Schraven B (2008). Regulation of T Cell Homeostasis by the Transmembrane Adaptor Protein SIT.. J Immunol.

[8] Koelsch U, Schraven B, Simeoni L (2008). SIT and TRIM determine T cell fate in the thymus.. J Immunol.

[9] Birkenkamp KU, Coffer PJ (2003). FOXO transcription factors as regulators of immune homeostasis: molecules to die for?. J Immunol.

[10] Kerdiles YM, Beisner DR, Tinoco R, Dejean AS, Castrillon DH (2009). Foxo1 links homing and survival of naive T cells by regulating L-selectin, CCR7 and interleukin 7 receptor.. Nat Immunol.

[11] Horn J, Wang X, Reichardt P, Stradal TE, Warnecke N (2009). Src homology 2-domain containing leukocyte-specific phosphoprotein of 76 kDa is mandatory for TCR-mediated inside-out signaling, but dispensable for CXCR4-mediated LFA-1 activation, adhesion, and migration of T cells.. J Immunol.

[12] Kolsch U, Arndt B, Reinhold D, Lindquist JA, Juling N (2006). Normal T-cell development and immune functions in TRIM-deficient mice.. Mol Cell Biol.

[13] Marie-Cardine A, Kirchgessner H, Bruyns E, Shevchenko A, Mann M (1999). SHP2-interacting transmembrane adaptor protein (SIT), a novel disulfide-linked dimer regulating human T cell activation.. J Exp Med.

[14] Charvet C, Canonigo AJ, Becart S, Maurer U, Miletic AV (2006). Vav1 promotes T cell cycle progression by linking TCR/CD28 costimulation to FOXO1 and p27kip1 expression.. J Immunol.

[15] Freeburn RW, Wright KL, Burgess SJ, Astoul E, Cantrell DA (2002). Evidence that SHIP-1 contributes to phosphatidylinositol 3,4,5-trisphosphate metabolism in T lymphocytes and can regulate novel phosphoinositide 3-kinase effectors.. J Immunol.

[16] Shan X, Czar MJ, Bunnell SC, Liu P, Liu Y (2000). Deficiency of PTEN in Jurkat T cells causes constitutive localization of Itk to the plasma membrane and hyperresponsiveness to CD3 stimulation.. Mol Cell Biol.

[17] Jang IK, Zhang J, Chiang YJ, Kole HK, Cronshaw DG (2010). Grb2 functions at the top of the T-cell antigen receptor-induced tyrosine kinase cascade to control thymic selection.. Proc Natl Acad Sci U S A.

[18] Pfrepper KI, Marie-Cardine A, Simeoni L, Kuramitsu Y, Leo A (2001). Structural and functional dissection of the cytoplasmic domain of the transmembrane adaptor protein SIT (SHP2-interacting transmembrane adaptor protein).. Eur J Immunol.

[19] Lin L, Hron JD, Peng SL (2004). Regulation of NF-kappaB, Th activation, and autoinflammation by the forkhead transcription factor Foxo3a.. Immunity.

[20] Herzog S, Hug E, Meixlsperger S, Paik JH, DePinho RA (2008). SLP-65 regulates immunoglobulin light chain gene recombination through the PI(3)K-PKB-Foxo pathway.. Nat Immunol.

